# The Complete Mitochondrial Genome and Phylogenetic Position of the Pacific Spiny Dogfish *Squalus suckleyi* (Squaliformes: Squalidae)

**DOI:** 10.1002/ece3.74113

**Published:** 2026-07-28

**Authors:** Laure Inçaby, Sabrina Haney, Michael Phelps, Catherine S. Jones, Marcela Uliano‐Silva, Galice Hoarau, Leslie R. Noble

**Affiliations:** ^1^ Faculty of Biosciences and Aquaculture Nord University Bodø Norway; ^2^ Department of Animal Science Washington State University Pullman WA USA; ^3^ School of Biological Sciences University of Aberdeen Aberdeen UK; ^4^ Tree of Life Wellcome Sanger Institute Hinxton UK

**Keywords:** comparative mitogenomics, elasmobranch, genomic resources, mitogenome, North Pacific

## Abstract

The Pacific spiny dogfish, *Squalus suckleyi* (Girard, 1854), is a benthopelagic small shark widely distributed across the North Pacific, caught in both commercial and bycatch fisheries throughout its range. Here we report the first mitochondrial genome of *Squalus suckleyi* , assembled from PacBio high‐fidelity sequencing reads. The mitogenome (GenBank accession PZ086324) is 16,738 base pairs long and comprises the typical set of 37 mitochondrial genes found in vertebrates, including 13 protein‐coding genes, 22 transfer RNA genes, two ribosomal RNA genes and a control region. The overall nucleotide composition is AT‐biased (A + T content of 61.17%), consistent with other elasmobranchs. Maximum‐likelihood phylogenetic analysis based on the 13 protein‐coding genes recovered *S. suckleyi* as sister to *S. acanthias* with maximum ultrafast bootstrap support. Pairwise non‐synonymous to synonymous substitution rate ratios between *S. suckleyi* and *S. acanthias* ranged from 0 to 0.051 across all protein‐coding genes, suggesting strong purifying selection on mitochondrial function in this recently diverged species pair. This mitogenome provides a high‐quality genomic resource to support phylogeographic, evolutionary and management‐oriented studies of *Squalus suckleyi* and more broadly the Squaliformes.

## Graphical abstract

The Pacific spiny dogfish ( *Squalus suckleyi* ) is a North Pacific shark of conservation consern that until now lacked a complete mitochondrial genome. We report its first mitogenome (16,738 bp; 37 genes), assembled from PacBio HiFi reads and recover it as sister to *S. acanthias* with strong purifying selection across all protein‐coding genes, providing a useful genomic resource for future evolutionary, comparative and phylogeographic studies in *Squalus* and across Squaliformes.

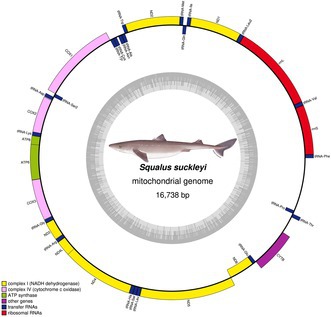

## 1 Introduction

The Pacific spiny dogfish, *Squalus suckleyi* (Girard, 1854), is a small benthopelagic shark distributed across the North Pacific, from the West Coast of North America to the coasts of Japan and the Korean peninsula (Ebert et al. 2010). *Squalus suckleyi* occurs from the intertidal zone down to approximately 1200 m depth, typically in cool waters of 7°C to 15°C, and undertakes seasonal latitudinal and depth migrations to remain within this thermal range (Andrews and Harvey 2013). As a generalist mesopredator, *S. suckleyi* feeds mostly on shrimps, cephalopods and small schooling fish, with salmon and rockfish forming only a minor part of its diet (Tribuzio et al. 2017).

For most of the twentieth century, *S. suckleyi* was the subject of a long‐standing taxonomic debate. Originally described from Puget Sound by Girard (1854), it was subsequently synonymised with the North Atlantic *Squalus acanthias* (Linnaeus, 1758) despite differences in life‐history traits, meristic characters and molecular markers between the two species. Combined morphometric and CO1 barcoding evidence (Ebert et al. 2010) together with concordant data from nuclear microsatellites and mitochondrial ND2 (Veríssimo et al. 2010) eventually resurrected *S. suckleyi* as a distinct evolutionary entity restricted to the North Pacific.

*Squalus suckleyi* is subject to targeted and bycatch fisheries across its range. Life‐history traits typical of K‐selected elasmobranchs, including a maximum size of approximately 130 cm total length and an estimated lifespan of up to 90 years (Tribuzio and Kruse 2012), render populations especially vulnerable to fishing pressure. While currently listed as “Least Concern” by the IUCN Red List of Threatened Species (Bigman et al. 2016), the species has recently been shown to have undergone a 51% coastwide population decline across the Northeast Pacific over the last 20 years (Davidson et al. 2026).

Mitochondrial genomes are widely used in shark systematics and phylogenetics (e.g., Zhu et al. 2017; Skufca and Baeza 2025) due to their generally conserved structure, maternal inheritance, small genome size, faster rate of sequence evolution than the nuclear genome (Martin et al. 1992), high copy number and low rate of intermolecular genetic recombination (Birky et al. 1989; Hoarau et al. 2002; Galtier et al. 2009). Within Squalidae, complete mitochondrial genomes are already available for *S. acanthias* (Rasmussen and Arnason 1999), *S. blainville* (Kousteni et al. 2021), *S. brevirostris* (Zhang et al. 2019), *S. clarkae* (PV651710, unpublished), *S. cubensis* (Skufca and Baeza 2025), *S. formosus* (Chen et al. 2016) and *S. montalbani* (Kemper and Naylor 2016). Here, we present the complete mitochondrial genome of *S. suckleyi*, assembled from PacBio HiFi reads and assess its gene content, nucleotide and structural composition and use it to determine the phylogenetic position of *S. suckleyi* within the Squalidae. This mitogenome is intended to facilitate ongoing and future evolutionary, comparative and phylogeographic studies within *Squalus* and across the Squaliformes.

## 2 Materials and Methods

A female *Squalus suckleyi* was collected in Blaine, Washington, USA (48.9917° N, 122.765° W) by Sabrina Haney; specimen metadata are available under BioSample SAMN47447513. A blood sample from this individual was accessioned at the Burke Museum of Natural History and Culture, University of Washington, Seattle, WA, USA (voucher UW 207257; sample identifier fSquSuc5.BL3, stored in 100% ethanol at −80°C). The corresponding mitogenome was deposited on GenBank under isolate name sSquSuc3 (accession PZ086324); fSquSuc5.BL3 and sSquSuc3 refer to the same individual under different internal naming conventions. High molecular weight DNA was extracted by the Vertebrate Genomes Project (VGP) Phase 1 consortium from 20 μL of blood using the Nanobind PanDNA kit (PacBio PN 103–260‐000), quantified with a Qubit 3 Fluorometer and quality‐assessed using an Agilent Femto Pulse instrument. The PacBio HiFi sequencing reads generated by the same consortium were retrieved from NCBI Sequence Read Archive (SRR33592372; BioProject PRJNA1263556) (Larivière et al. 2024). As this study was based entirely on pre‐existing, publicly available sequencing data, and on a previously accessioned voucher specimen, no additional animal sampling or handling was performed by the authors and no ethical clearance number was required.

Mitochondrial assembly and circularization were performed using MitoHiFi (v3.2.3) (Uliano‐Silva et al. 2023), using a recent *S. acanthias* mitochondrial genome (PX718978, unpublished) as the closely related reference. To confirm that the choice of reference did not influence the assembly, we also performed an independent run using the classical *S. acanthias* reference (NC_002012, Rasmussen and Arnason 1999) and recovered an identical mitogenome sequence. Gene annotation was conducted within the MitoHiFi pipeline using MitoFinder (Allio et al. 2020) and ARWEN (Laslett and Canbäck 2008). The resulting mitochondrial genome was manually inspected to confirm circularity and gene completeness, and a graphical genome map was generated using OGDRAW (v1.3.1) (Greiner et al. 2019). All predicted tRNA loci were identified using tRNAscan‐SE (v2.0.12) in vertebrate mitochondrial mode (Chan et al. 2021); and visualised with RNAplot from the ViennaRNA package (v2.7.2) (Hofacker et al. 1994; Lorenz et al. 2011) (Figure S1).

To assess the phylogenetic position of *S. suckleyi* , we compared our newly assembled mitochondrial genome (PZ086324) with complete mitochondrial genomes of closely related species retrieved from GenBank, including *S. acanthias* (NC_002012, Rasmussen and Arnason 1999), *S. montalbani* (KT459334, Kemper and Naylor 2016), *S. formosus* (KU951280, Chen et al. 2016), *S. brevirostris* (KY111436, Zhang et al. 2019), *S. blainville* (NC_059940, Kousteni et al. 2021), *S. cubensis* (OP056876, Skufca and Baeza 2025) and *S. clarkae* (PV651710, unpublished). In addition, two recently deposited *S. acanthias* mitogenomes (PV651714 and PX718978, both unpublished) were added to test reciprocal monophyly between *S. acanthias* and *S. suckleyi* at the mitogenome level. *Centrophorus granulosus* (PX704710, unpublished) was selected as the outgroup as it belongs to the Squaliformes order but to a different family (Centrophoridae), providing an appropriate external lineage while remaining sufficiently close to avoid long‐branch artefacts. All mitochondrial genomes were downloaded as published and were not re‐annotated. For each species, the 13 mitochondrial protein‐coding genes (PCGs) were extracted and aligned independently using MAFFT (v7.520) under default parameters (Katoh et al. 2019; Kuraku et al. 2013). Individual alignments were concatenated into a single dataset of 11,446 base pairs. A partitioned maximum‐likelihood phylogenetic inference was conducted using IQ‐TREE (v3.0.1) (Wong et al. 2025), with one partition per gene and an edge‐linked‐proportional partition model; the best fitting nucleotide substitution model for each partition was selected by ModelFinder (Kalyaanamoorthy et al. 2017) under the Bayesian Information Criterion (BIC). Node support was assessed using 1000 ultrafast bootstrap replicates (UFBoot2) (Hoang et al. 2018) and 1000 SH‐aLRT replicates. The resulting tree was displayed in iTOL (v7.4.2) (Letunic and Bork 2021). To assess the robustness of the topology to the choice of inference method, we additionally reconstructed a distance‐based Minimum Evolution (ME) tree (Rzhetsky and Nei 1993) from the same concatenated alignment in MEGA12 (Kumar et al. 2024), using the Tamura‐Nei model with gamma‐distributed among site rate variation (5 categories) and a Close‐Neighbour‐Interchange search from a Neighbour‐Joining starting tree. The partial deletion option was applied with a 95% site coverage cutoff (11,089 positions retained), and note support was estimated from 1000 bootstrap replicates (Figure S3).

Relative synonymous codon usage (RSCU) values for the 13 protein‐coding genes were computed using the vertebrate mitochondrial genetic code (NCBI translation table 2). To examine selective pressure on protein‐coding genes, non‐synonymous (Ka) and synonymous (Ks) substitution rates and their ratios (Ka/Ks) were calculated between the *S. suckleyi* mitogenome (this study) and its closest available relative *S. acanthias* (PV651714) using the Nei‐Gojobori method (Nei and Gojobori 1986) with Jukes‐Cantor correction and pathway averaging across the vertebrate mitochondrial genetic code. Pairwise nucleotide identity between *S. suckleyi* and other Squalidae mitogenomes was additionally computed across five mitochondrial regions commonly used in shark barcoding and phylogeography (ND2, COX1, CYTB, 12S rRNA and the D‐loop) from per‐gene MAFFT alignments (Table S5).

Heteroplasmy was quantified by aligning the PacBio HiFi reads (SRR33592372; BioProject PRJNA1263556) to the assembled mitogenome with minimap2 (v2.31) (Li 2018) using the HiFi preset. Per‐position allele counts at primary alignments (MAPQ≥ 20) were extracted with bcftools mpileup (v1.23.1) (Danecek et al. 2021). Candidate heteroplasmic sites were defined as positions with minor‐allele frequency (MAF) ≥ 1%, depth ≥ 30 × and minor‐allele count ≥ 3. Homopolymer‐tract indels and sites with strand‐biased alternative reads were excluded. To identify potential nuclear mitochondrial DNA segments (NUMTs), a 500 bp window around each remaining candidate was aligned to the *S. suckleyi* nuclear assembly (GCA_050613895.1) with minimap2 (v2.31) (Li 2018), candidates aligning at ≥ 80% identify over ≥ 100 bp were excluded as potential NUMTs.

## 3 Results and Discussion

**FIGURE 1:**
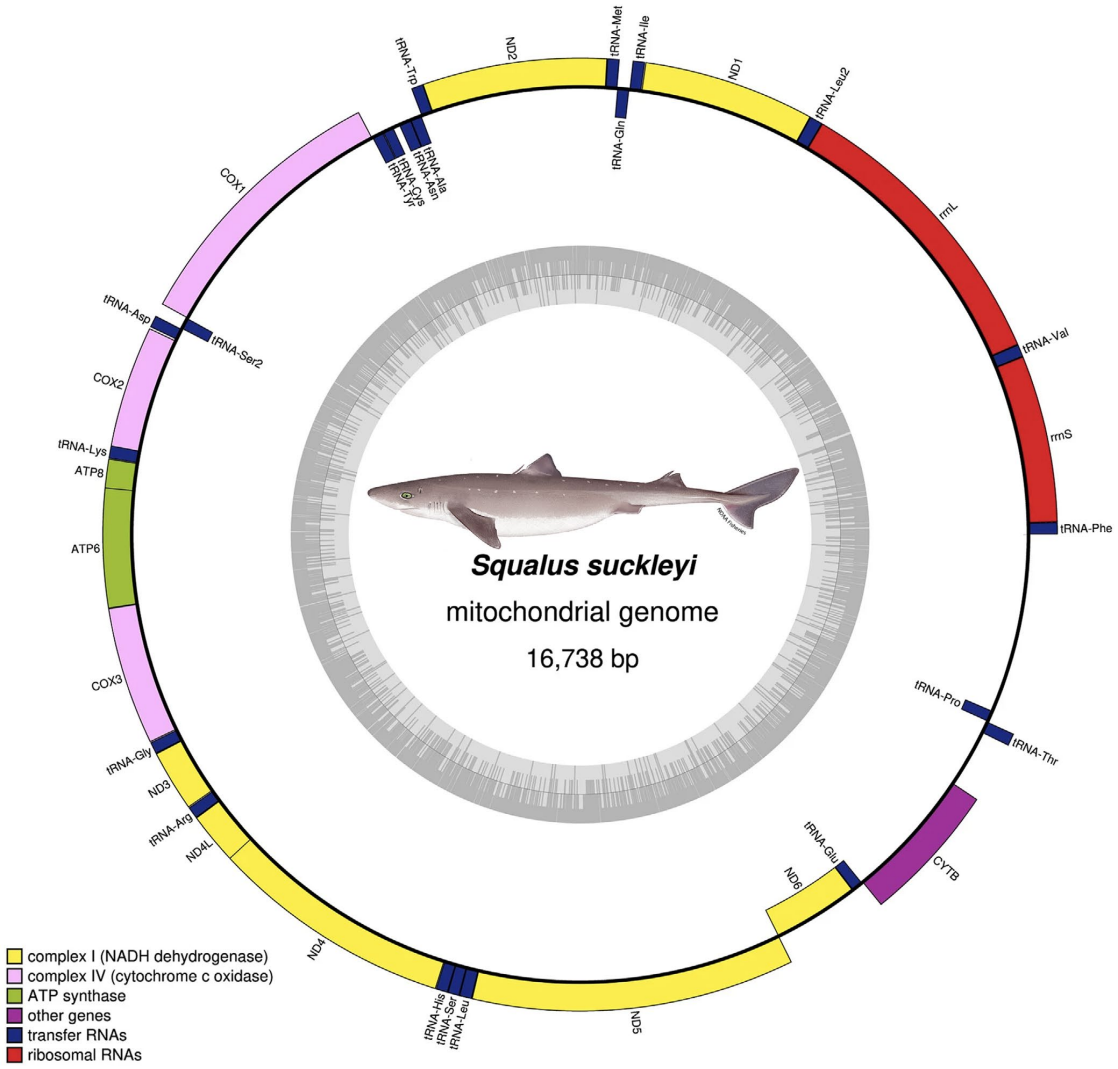
Circular mitochondrial genome of *Squalus suckleyi* . Genes encoded on the heavy (H, external) and light (L, internal) strands are indicated. Illustration of the studied species by NOAA Fisheries, used with permission.

The complete mitochondrial genome of *Squalus suckleyi* assembled in this study was 16,738 base pairs long. Read coverage across the genome was high, with a mean coverage depth of 150.8 × (Figure S1), indicating homogenous sequencing depth and supporting the accuracy of the assembly. As expected for vertebrate mitogenomes, it contains the standard set of 37 mitochondrial genes (Table 1) with 13 protein‐coding genes (PCGs), 22 tRNA genes and 2 ribosomal RNA (rRNA) genes (12S and 16S), as well as a non‐coding control region (Figure 1). Most genes are encoded on the heavy strand, while ND6 and several tRNA genes are located on the light strand. Gene order and orientation are consistent with those reported for other *Squalus* species (Skufca and Baeza 2025), and other vertebrate mitogenomes (Boore 1999), with no rearrangements, duplications or translocations detected. The overall nucleotide composition is biased towards adenine and thymine, with base frequencies of A: 30.80%, T: 30.37%, G: 14.31% and C: 24.51%, resulting in an A + T content of 61.17% and G + C content of 38.83%, as commonly observed in most vertebrate mitogenomes (Table S1). All 22 tRNA genes were detected and could be folded into canonical cloverleaf secondary structures (Figure S2), with the exception of trnS1 (Ser1, AGY), which lacks the dihydrouridine (DHU) arm as reported in other *Squalus* and elasmobranch species (Kousteni et al. 2021; Skufca and Baeza 2025). Both the total genome and the tRNA lengths fall within the range reported for other *Squalus* species (approximately 16.6–16.8 kb) (Table S1).

**TABLE 1:** Organisation of the complete mitochondrial genome of *Squalus suckleyi* (PZ086324).

Gene	Strand	Start	End	Length (bp)	Start codon	Stop codon	Anticodon	Intergenic (bp)
tRNA‐Phe (F)	H	1	69	69			GAA	
rrnS (12S)	H	70	1020	951				0
tRNA‐Val (V)	H	1021	1092	72			TAC	0
rrnL (16S)	H	1093	2768	1676				0
tRNA‐Leu2 (L2, UUR)	H	2769	2843	75			TAA	0
ND1	H	2844	3818	975	ATG	TAA		0
tRNA‐Ile (I)	H	3821	3890	70			GAT	2
tRNA‐Gln (Q)	L	3892	3963	72			TTG	1
tRNA‐Met (M)	H	3964	4032	69			CAT	0
ND2	H	4033	5079	1047	ATG	TAA		0
tRNA‐Trp (W)	H	5079	5147	69			TCA	−1
tRNA‐Ala (A)	L	5149	5217	69			TGC	1
tRNA‐Asn (N)	L	5218	5291	74			GTT	0
tRNA‐Cys (C)	L	5330	5396	67			GCA	38
tRNA‐Tyr (Y)	L	5398	5467	70			GTA	1
COX1	H	5469	7025	1557	GTG	TAA		1
tRNA‐Ser2 (S2, UCN)	L	7026	7096	71			TGA	0
tRNA‐Asp (D)	H	7099	7168	70			GTC	2
COX2	H	7177	7875	699	ATG	AGA		8
tRNA‐Lys (K)	H	7868	7941	74			TTT	−8
ATP8	H	7943	8110	168	ATG	TAA		1
ATP6	H	8101	8784	684	ATG	TAA		−10
COX3	H	8784	9569	786	ATG	TAA		−1
tRNA‐Gly (G)	H	9572	9641	70			TCC	2
ND3	H	9642	9992	351	ATG	TAA		0
tRNA‐Arg (R)	H	9995	10,064	70			TCG	2
ND4L	H	10,065	10,361	297	ATG	TAA		0
ND4	H	10,355	11,734	1380	ATG	T‐‐^a^		−7
tRNA‐His (H)	H	11,736	11,804	69			GTG	1
tRNA‐Ser1 (S1, AGY)	H	11,805	11,871	67			GCT	0
tRNA‐Leu1 (L1, CUN)	H	11,872	11,943	72			TAG	0
ND5	H	11,944	13,776	1833	ATG	TAA		0
ND6	L	13,773	14,294	522	ATG	TAG		−4
tRNA‐Glu (E)	L	14,295	14,364	70			TTC	0
CYTB	H	14,369	15,514	1146	ATG	TAA		4
tRNA‐Thr (T)	H	15,516	15,588	73			TGT	1
tRNA‐Pro (P)	L	15,591	15,659	69			TGG	2
Control region (D‐loop)		15,660	16,738	1079				0

*Note:* Intergenic distance: Number of bases between the end of the preceding gene and the start of the listed gene; negative values indicate overlap.

Abbreviations: H, heavy strand; L, light strand.

^a^ Incomplete stop codon presumed completed by post‐transcriptional polyadenylation.

The 13 PCGs span 11,433 bp using vertebrate mitochondrial genetic code (Table 1). Twelve PCGs initiate with ATG, while *COX1* initiates with GTG, an alternative start codon previously reported in *S. cubensis* and other Squalidae (Skufca and Baeza 2025). Ten PCGs terminate with the canonical TAA stop codon, COX2 with AGA and ND6 with TAG; while ND4 terminates with an incomplete stop codon, presumed completed by post‐transcriptional polyadenylation. The control region (D‐loop) spans 1079 bp between *trnP* and *trnF*, identical in length to that of *S. acanthias* (Table 1). Across all 3815 codons (Table S2), relative synonymous codon usage (RSCU) analysis revealed a clear bias towards AT‐rich codons. Fourteen codons were preferentially used (RSCU ≥ 1.5), with the strongest biases observed for CGA (Arg, RSCU = 2.19), TTA (Leu, RSCU = 2.04), TCA (Ser, RSCU = 1.94), CAA (Gln, RSCU = 1.86) and AAA (Lys, RSCU = 1.83), paralleling the codon usage pattern reported for *S. cubensis* (Skufca and Baeza 2025). Pairwise non‐synonymous to synonymous substitution rate ratios (Ka/Ks) between *S. suckleyi* and *S. acanthias* (PV651714) ranged from 0 to 0.051 across the 13 PCGs (Table S3), with 125 synonymous and only 8 non‐synonymous substitutions over 11,433 bp. All Ka/Ks values were substantially below unity, consistent with strong purifying selection on protein function in this recently diverged sister‐species pair (Hurst 2002; Yang et al. 2000).

**FIGURE 2:**
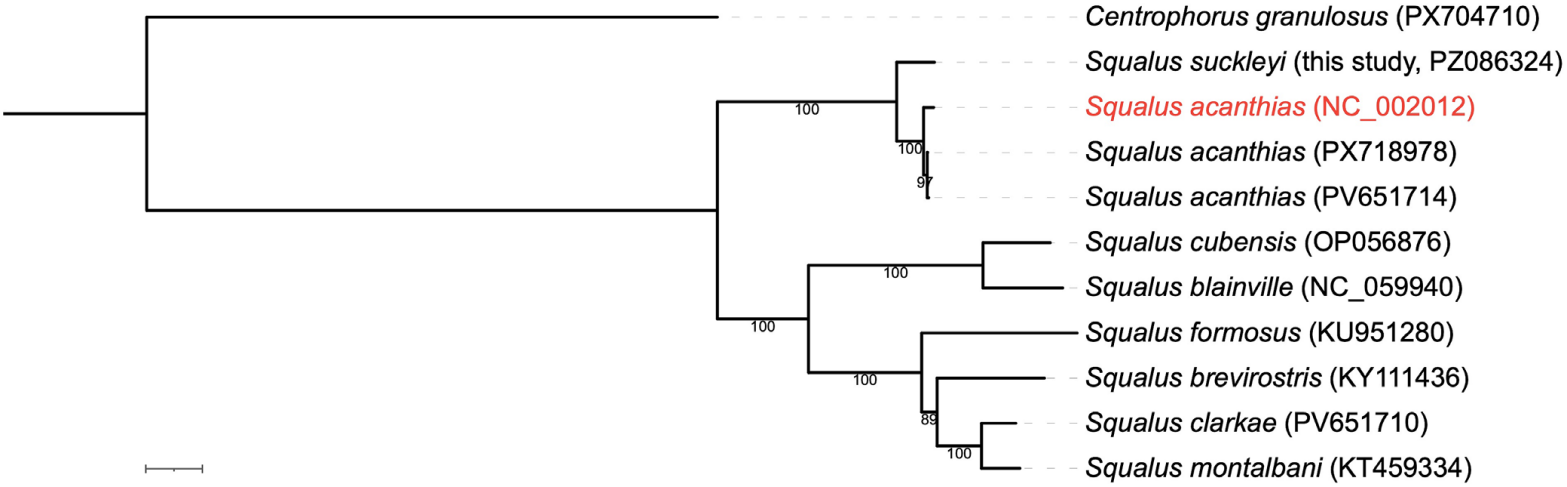
Maximum‐likelihood phylogenetic tree based on the 13 mitochondrial protein‐coding genes (concatenated 11,446 bp matrix, partitioned by gene) showing the position of *Squalus suckleyi* among related taxa. Numbers at nodes indicate ultrafast bootstrap support. The tree was rooted with *Centrophorus granulosus* . GenBank accession numbers are given after species names.

To determine the phylogenetic position of *S. suckleyi* , a maximum‐likelihood (ML) analysis was conducted using a concatenated nucleotide alignment of all 13 mitochondrial PCGs (11,446 bp). The resulting tree recovered *Squalus suckleyi* as sister to *Squalus acanthias* with maximum ultrafast bootstrap support (UFBoot = 100), forming a well‐supported clade distinct from other species including *S. montalbani* , *S. formosus* , *S. brevirostris* , *S. blainville* , *S. cubensis* and *S. clarkae* (Figure 2). This sister‐species relationship was also recovered by a complementary Minimum Evolution (ME) analysis (with maximum bootstrap support) (Figure S3). The outgroup taxon ( *Centrophorus granulosus* ) was clearly separated from the ingroup, and no topological instability was observed. All major nodes received high to maximum ultrafast bootstrap support (UFBoot≥ 92).

Together, these analyses confirm the sister‐species relationship between *S. suckleyi* and *S. acanthias* based on the 13 mitochondrial protein‐coding genes, with reciprocal monophyly recovered between mitogenomes included in the phylogeny (Figure 2) and further reinforced by the Minimum Evolution analysis (Figure S3). The very low Ka/Ks values across all PCGs (0–0.051) are consistent with this recent divergence and indicate strong purifying selection on mitochondrial function in both lineages, in line with the pattern of strong purifying selection consistently reported for Squalidae mitogenomes and sharks in general (Skufca and Baeza 2025; Zhu et al. 2017). Identical control region length (1079 bp) and identical total mitogenome length (16,738 bp) of *S. suckleyi* and *S. acanthias* further support their close evolutionary relationship, while nucleotide‐level differences captured across the PCGs provide reliable molecular markers for distinguishing these sister species in downstream applications such as stock identification and phylogeographic surveys. Pairwise identities between *S. suckleyi* and *S. acanthias* ranged from 98.52% to 99.89% across ND2, COX1, CYTB, 12 S rRNA and the D‐loop, compared to 90.40%–98.52% between *S. suckleyi* and other *Squalus* sharks (Table S5).

Only two heteroplasmic positions exceeded the 1% MAF threshold after filtering: position 3789 in ND1 (codon 316, G➔A, MAF = 8.9%, Ala➔Thr) and position 9705 in ND3 (codon 22, G➔A, Ala➔Thr) (Table S4). Both encode non‐synonymous substitutions at first codon positions, with balanced strand representation among the alternative reads. No heteroplasmy was detected in the control region. The alternative allele at position 9705, supported by 65 independent reads, was absent from the other 9 Squalidae mitogenomes used in the phylogenetic analysis.

## 4 Conclusion

We report the complete mitochondrial genome of *Squalus suckleyi* , which is 16,738 bp and exhibits the conserved gene content and organisation characteristic of vertebrate mitogenomes. The genome shows no structural rearrangements and presents nucleotide composition patterns consistent with other elasmobranchs. Phylogenetic analysis based on 13 PCGs recovers *S. suckleyi* as sister to *S. acanthias* . The availability of the *S. suckleyi* mitochondrial genome provides an important genomic resource for future evolutionary, comparative and phylogeographic studies, and will facilitate further investigations of species boundaries and historical divergence within the Squaliformes.

## Author Contributions

**Laure Inçaby:** conceptualization (lead), formal analysis (lead), investigation (lead), methodology (lead), validation (lead), visualization (lead), writing – original draft (lead), writing – review and editing (equal). **Sabrina Haney:** resources (equal), writing – review and editing (equal). **Michael Phelps:** resources (equal), writing – review and editing (equal). **Catherine S. Jones:** supervision (equal), writing – review and editing (equal). **Marcela Uliano‐Silva:** project administration (equal), software (lead), supervision (equal), writing – review and editing (equal). **Galice Hoarau:** project administration (equal), supervision (equal), writing – review and editing (equal). **Leslie R. Noble:** project administration (equal), supervision (equal), writing – review and editing (equal).

## Funding

L.I. was supported by Nord Universitet (224000‐197).

## Ethics Statement

This study was based on publicly available sequencing data (SRR33592372, BioProject PRJNA1263556) generated by the Vertebrate Genome Project, and on a voucher specimen (UW 207257) previously accessioned at the Burke Museum of Natural History and Culture (University of Washington). The original blood sample was collected non‐lethally. No additional animal sampling, handling or experimentation was conducted by the authors of this study, and no ethical clearance number was therefore required for this work.

## Conflicts of Interest

The authors declare no conflicts of interest.

## Supporting information


**Figure S1:** Coverage depth figure obtained by MitoHiFi (Uliano‐Silva et al. 2023) for the mitochondrial genome of *Squalus suckleyi* .
**Figure S2:** 22 tRNAs secondary structures in the mitochondrial genome of *Squalus suckleyi* .
**Figure S3:** Minimum Evolution (ME) phylogenetic tree based on the concatenated 13 mitochondrial protein‐coding genes (11,089 positions retained after partial deletion), showing the position of *Squalus suckleyi* among related taxa. Numbers at node indicate bootstrap support. The tree was rooted with *Centrophorus granulosus* . GenBank accession numbers are given after species names.
**Table S1:** Comparative mitogenome statistics across Squalidae and the outgroup *Centrophorus granulosus* . AT‐skew = (A‐T)/(A + T); GC‐skew = (G‐C)/(G + C). D‐loop length given where annotated; NA = not annotated as a discrete feature in the GenBank record.
**Table S2:** Codon usage and relative synonymous codon usage (RSCU) values for the 13 protein‐coding genes of *Squalus suckleyi* (3815 codons total) under the vertebrate mitochondrial genetic code (NCBI translation table 2). Codons with RSCU ≥ 1.5 are shown in bold.
**Table S3:** Pairwise non‐synonymous (Ka) and synonymous (Ks) substitution rates between the 13 mitochondrial protein‐coding genes of *Squalus suckleyi* (PZ086324) and *Squalus acanthias* (PV651714). Calculated by the Nei and Gojobori 1986 method with Jukes‐Cantor correction. S_sites, synonymous sites: N_sites, non‐synonymous sites; Sd, synonymous differences, Nd, non‐synonymous differences.
**Table S4:** Heteroplasmic positions detected in the *Squalus suckleyi* mitogenome (PZ086324) from PacBio HiFi reads (SRR33592372). MAF, minor‐allele frequency.
**Table S5:** Pairwise sequence identity (%) across five mitochondrial regions commonly used in shark DNA barcoding (ND2, COX1, CYTB) and phylogeography (12S rRNA, D‐loop) for the 10 *Squalus* mitogenomes analysed in this study. Values computed from per‐gene MAFFT alignments.

## Data Availability

The GenBank accession number for the complete mitochondrial genome sequence of *Squalus suckleyi* is PZ086324. The BioProject, BioSample and SRA numbers are PRJNA1263556, SAMN47447513 and SRR33592372, respectively.
