# Supplementary figures and images for: The Complete Mitochondrial Genome and Phylogenetic Position of the Pacific Spiny Dogfish Squalus suckleyi (Squaliformes: Squalidae)
Source: Ecol Evol. 2026 Jul 28;16(8):e74113. doi: 10.1002/ece3.74113 (PMC13415751; doi:10.1002/ece3.74113)

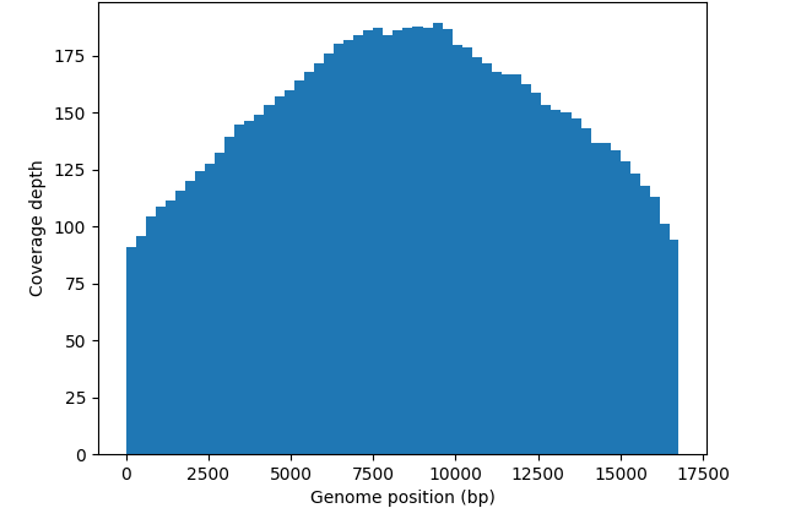

Supplement: Supplementary file 1 — Figure S1: Coverage depth figure obtained by MitoHiFi (Uliano‐Silva et al. 2023) for the mitochondrial genome of Squalus suckleyi . Figure S2: 22 tRNAs secondary structures in the mitochondrial genome of Squalus suckleyi . Figure S3: Minimum Evolution (ME) phylogenetic tree based on the concatenated 13 mitochondrial protein‐coding genes (11,089 positions retained after partial deletion), showing the position of Squalus suckleyi among related taxa. Numbers at node indicate bootstrap support. The tree was rooted with Centrophorus granulosus . GenBank accession numbers are given after species names. Table S1: Comparative mitogenome statistics across Squalidae and the outgroup Centrophorus granulosus . AT‐skew = (A‐T)/(A + T); GC‐skew = (G‐C)/(G + C). D‐loop length given where annotated; NA = not annotated as a discrete feature in the GenBank record. Table S2: Codon usage and relative synonymous codon usage (RSCU) values for the 13 protein‐coding genes of Squalus suckleyi (3815 codons total) under the vertebrate mitochondrial genetic code (NCBI translation table 2). Codons with RSCU ≥ 1.5 are shown in bold. Table S3: Pairwise non‐synonymous (Ka) and synonymous (Ks) substitution rates between the 13 mitochondrial protein‐coding genes of Squalus suckleyi (PZ086324) and Squalus acanthias (PV651714). Calculated by the Nei and Gojobori 1986 method with Jukes‐Cantor correction. S_sites, synonymous sites: N_sites, non‐synonymous sites; Sd, synonymous differences, Nd, non‐synonymous differences. Table S4: Heteroplasmic positions detected in the Squalus suckleyi mitogenome (PZ086324) from PacBio HiFi reads (SRR33592372). MAF, minor‐allele frequency. Table S5: Pairwise sequence identity (%) across five mitochondrial regions commonly used in shark DNA barcoding (ND2, COX1, CYTB) and phylogeography (12S rRNA, D‐loop) for the 10 Squalus mitogenomes analysed in this study. Values computed from per‐gene MAFFT alignments. [file ECE3-16-e74113-s001.zip › ece374113-sup-0001-FigureS1.png]

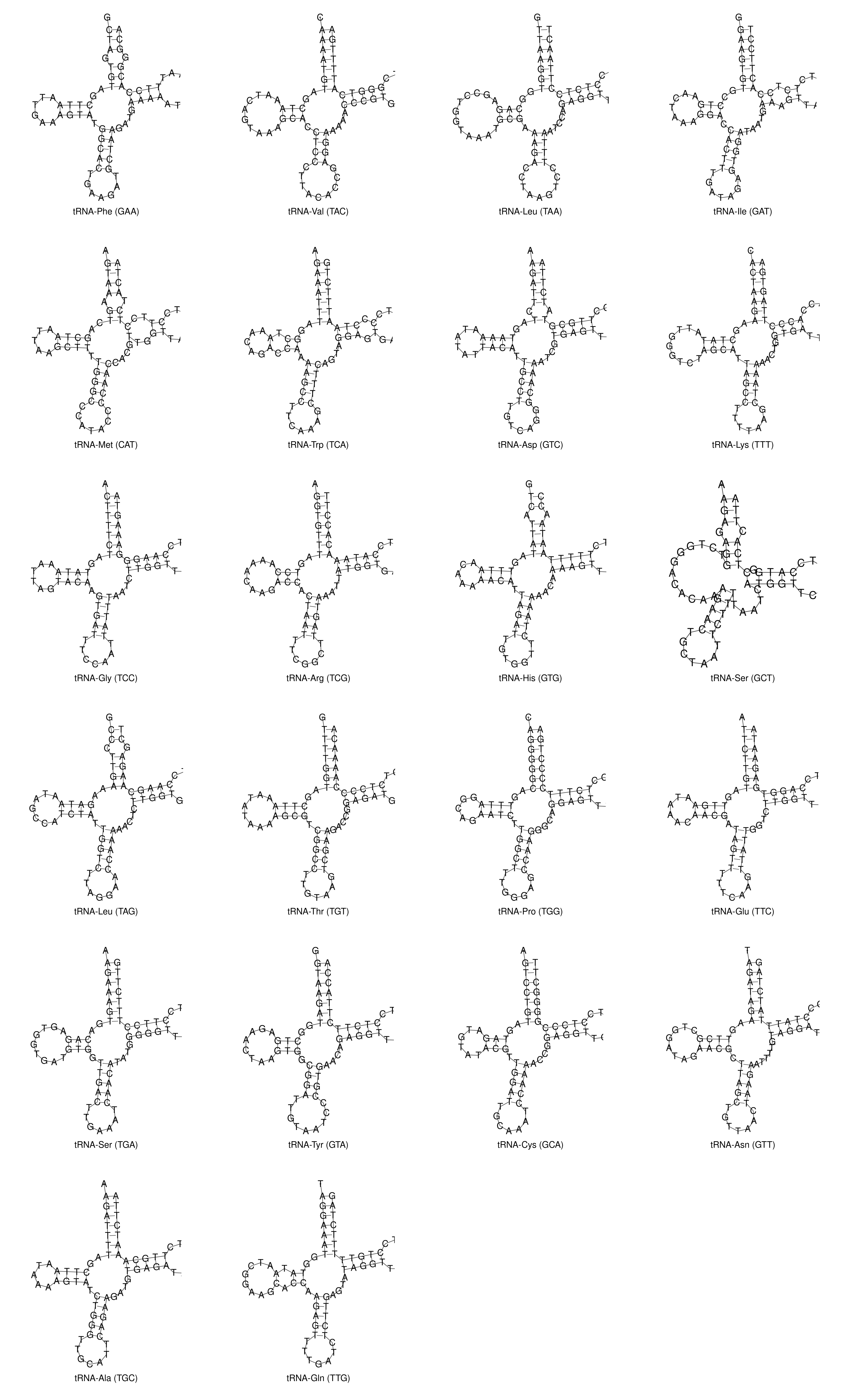

Supplement: Supplementary file 1 — Figure S1: Coverage depth figure obtained by MitoHiFi (Uliano‐Silva et al. 2023) for the mitochondrial genome of Squalus suckleyi . Figure S2: 22 tRNAs secondary structures in the mitochondrial genome of Squalus suckleyi . Figure S3: Minimum Evolution (ME) phylogenetic tree based on the concatenated 13 mitochondrial protein‐coding genes (11,089 positions retained after partial deletion), showing the position of Squalus suckleyi among related taxa. Numbers at node indicate bootstrap support. The tree was rooted with Centrophorus granulosus . GenBank accession numbers are given after species names. Table S1: Comparative mitogenome statistics across Squalidae and the outgroup Centrophorus granulosus . AT‐skew = (A‐T)/(A + T); GC‐skew = (G‐C)/(G + C). D‐loop length given where annotated; NA = not annotated as a discrete feature in the GenBank record. Table S2: Codon usage and relative synonymous codon usage (RSCU) values for the 13 protein‐coding genes of Squalus suckleyi (3815 codons total) under the vertebrate mitochondrial genetic code (NCBI translation table 2). Codons with RSCU ≥ 1.5 are shown in bold. Table S3: Pairwise non‐synonymous (Ka) and synonymous (Ks) substitution rates between the 13 mitochondrial protein‐coding genes of Squalus suckleyi (PZ086324) and Squalus acanthias (PV651714). Calculated by the Nei and Gojobori 1986 method with Jukes‐Cantor correction. S_sites, synonymous sites: N_sites, non‐synonymous sites; Sd, synonymous differences, Nd, non‐synonymous differences. Table S4: Heteroplasmic positions detected in the Squalus suckleyi mitogenome (PZ086324) from PacBio HiFi reads (SRR33592372). MAF, minor‐allele frequency. Table S5: Pairwise sequence identity (%) across five mitochondrial regions commonly used in shark DNA barcoding (ND2, COX1, CYTB) and phylogeography (12S rRNA, D‐loop) for the 10 Squalus mitogenomes analysed in this study. Values computed from per‐gene MAFFT alignments. [file ECE3-16-e74113-s001.zip › ece374113-sup-0002-FigureS2.png]

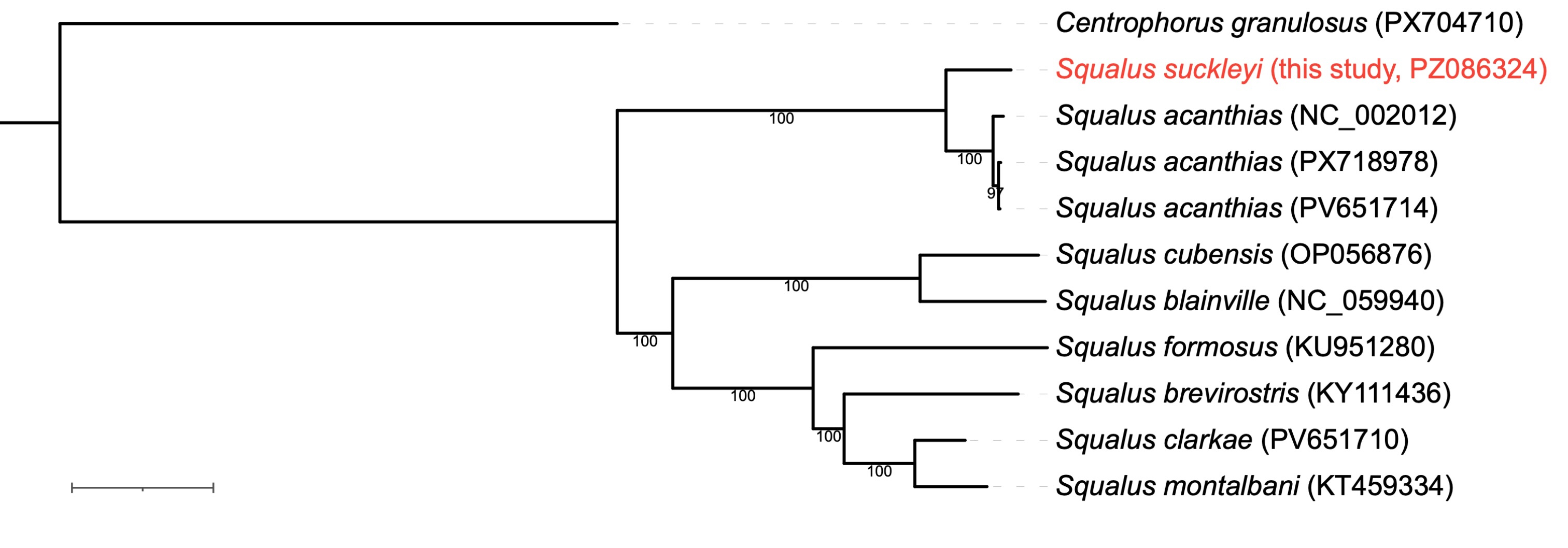

Supplement: Supplementary file 1 — Figure S1: Coverage depth figure obtained by MitoHiFi (Uliano‐Silva et al. 2023) for the mitochondrial genome of Squalus suckleyi . Figure S2: 22 tRNAs secondary structures in the mitochondrial genome of Squalus suckleyi . Figure S3: Minimum Evolution (ME) phylogenetic tree based on the concatenated 13 mitochondrial protein‐coding genes (11,089 positions retained after partial deletion), showing the position of Squalus suckleyi among related taxa. Numbers at node indicate bootstrap support. The tree was rooted with Centrophorus granulosus . GenBank accession numbers are given after species names. Table S1: Comparative mitogenome statistics across Squalidae and the outgroup Centrophorus granulosus . AT‐skew = (A‐T)/(A + T); GC‐skew = (G‐C)/(G + C). D‐loop length given where annotated; NA = not annotated as a discrete feature in the GenBank record. Table S2: Codon usage and relative synonymous codon usage (RSCU) values for the 13 protein‐coding genes of Squalus suckleyi (3815 codons total) under the vertebrate mitochondrial genetic code (NCBI translation table 2). Codons with RSCU ≥ 1.5 are shown in bold. Table S3: Pairwise non‐synonymous (Ka) and synonymous (Ks) substitution rates between the 13 mitochondrial protein‐coding genes of Squalus suckleyi (PZ086324) and Squalus acanthias (PV651714). Calculated by the Nei and Gojobori 1986 method with Jukes‐Cantor correction. S_sites, synonymous sites: N_sites, non‐synonymous sites; Sd, synonymous differences, Nd, non‐synonymous differences. Table S4: Heteroplasmic positions detected in the Squalus suckleyi mitogenome (PZ086324) from PacBio HiFi reads (SRR33592372). MAF, minor‐allele frequency. Table S5: Pairwise sequence identity (%) across five mitochondrial regions commonly used in shark DNA barcoding (ND2, COX1, CYTB) and phylogeography (12S rRNA, D‐loop) for the 10 Squalus mitogenomes analysed in this study. Values computed from per‐gene MAFFT alignments. [file ECE3-16-e74113-s001.zip › ece374113-sup-0003-FigureS3.jpg]
